# Nationwide trends in the incidence of tuberculosis among people with disabilities in Korea: a nationwide serial cross-sectional study

**DOI:** 10.4178/epih.e2022098

**Published:** 2022-10-28

**Authors:** Jinsoo Min, So Young Kim, Jong Eun Park, Yeon Yong Kim, Jong Hyock Park

**Affiliations:** 1 Division of Pulmonary and Critical Care Medicine, Department of Internal Medicine, Seoul St. Mary’s Hospital, College of Medicine, The Catholic University of Korea, Seoul, Korea; 2 Department of Public Health and Preventive Medicine, Chungbuk National University Hospital, Cheongju, Korea; 3 Institute of Health & Science Convergence, Chungbuk National University, Cheongju, Korea; 4 Big Data Steering Department, National Health Insurance Service, Wonju, Korea; 5 Drug Evaluation Department, National Institute of Food and Drug Safety Evaluation, Cheongju, Korea; 6 Department of Preventive Medicine, Chungbuk National University College of Medicine, Cheongju, Korea

**Keywords:** Intellectual disability, Mental disability, Incidence of tuberculosis, Risk, Vulnerable populations, Social determinants of health

## Abstract

**OBJECTIVES:**

Studies on the association between disabilities and tuberculosis (TB) are scarce. We aimed to assess the risk of active TB disease among people with disabilities.

**METHODS:**

We conducted a nationwide serial cross-sectional study using national registry linkage databases from 2008 to 2017. The crude and age-standardized and sex-standardized incidence rates of TB were analyzed for each year according to the presence, type, and severity of disabilities. The crude incidence rate and odds of developing TB disease were examined with a multivariable logistic regression model using data from 2017.

**RESULTS:**

The overall incidence of active TB decreased between 2008 and 2017. The age- and sex-standardized incidence rates of TB disease among people with disabilities were significantly higher than among those without disabilities throughout all observed years (p<0.001). As of 2017, the population with disabilities had a higher crude incidence rate of active TB disease than that without disabilities (119.9/100,000 vs. 48.5/100,000 person-years, p<0.001), regardless of sex, income level, and place of residence. Compared to those without disabilities, those with disabilities had higher odds of active TB (adjusted odds ratio [aOR], 1.19; 95% confidence interval [CI], 1.15 to 1.24). Individuals with mental disabilities (aOR, 1.51; 95% CI, 1.24 to1.84) had the highest odds of active TB incidence, followed by those with developmental disabilities (aOR, 1.30; 95% CI, 1.09 to 1.55).

**CONCLUSIONS:**

People with disabilities are at a greater risk of developing TB disease. Active screening and care for TB cases would be beneficial for people with disabilities.

## GRAPHICAL ABSTRACT

[Fig f2-epih-44-e2022098]

## INTRODUCTION

Tuberculosis (TB) is one of the most life-threatening infectious diseases and poses a significant risk to global health security. Despite being preventable and curable, TB remains a major cause of poor health and mortality. The World Health Organization (WHO) End TB Strategy seeks to prevent and control TB and recommends systematic screening for TB disease in high-risk groups as a central component of its first pillar [1]. The aim of screening is to detect TB disease early to minimize avoidable delays in diagnosis and treatment, thereby reducing the risk of unfavorable treatment outcomes, health sequelae, and adverse social and economic consequences of TB for individuals and their families.

People with disabilities are often at a greater risk for both communicable and chronic non-communicable diseases. Disabilities are associated with a diverse range of primary health conditions, accidents, injuries, and contextual factors, which lead to a wide range of functional limitations, including mobility, sensory, mental, and communication disabilities [2]. People with disabilities may be more susceptible to developing other chronic conditions because of their underlying conditions and the influence of behavioral risk factors, such as increased physical inactivity [3]. Disabilities may cause and reinforce poverty [4], which increases the risk of functional loss through malnutrition, poor healthcare access, and dangerous living, working, and traveling conditions [5]. These factors and conditions of disability often overlap with the risk factors for developing TB disease.

Disabilities are a global public health issue because they affect an estimated 15% of the world’s population [2,6], with an increasing prevalence due to an increase in chronic health conditions and population aging. Despite being considered a marginalized subpopulation, studies on the vulnerability of people with disabilities to developing TB disease are insufficient. If disabilities are a risk factor for TB, their burden may act as a significant barrier to global TB elimination. Our study aimed to elucidate the association between disabilities and active TB incidence and to compare trends in annual TB incidence between people with and without disabilities using a large nationwide cross-linked database.

## MATERIALS AND METHODS

### Study design and population

We conducted a nationwide serial cross-sectional study using linked national registry databases. We linked the National Disability Registration Database (NDRD) with the National Health Information Database (NHID) of Korea. The NHID is a public database on healthcare utilization, health screening, socio-demographic variables, and mortality for the entire Korean population, maintained by the Korean National Health Insurance Service [7]. From these data, we extracted information on socio-demographic variables.

The NDRD comprises data from the national registration system for people with disabilities, primarily for the provision of welfare benefits [8]. Registration requires the submission of appropriate and validated documentation to a local National Pension Service office. The paperwork includes appraised results of a disability diagnosis by a specialist physician in the corresponding field according to detailed criteria for the specific disability, as defined by the national disability registration system. From the NDRD, which covered 93.8% of the total population with disabilities in 2011 [9], we collected information on disabilities according to type and severity. The type and severity of disabilities were linked with the variables selected from the NHID using Korean personal identification numbers.

Data were anonymized by data holders before being accessed by the research team. We finally compiled cases involving people with disabilities between 2008 and 2017 using the NDRD (Supplementary Material 1).

### Definition of active tuberculosis disease and disabilities

The incidence of active TB disease was identified using the International Classification of Diseases (ICD), 10th revision, codes (A15-19), which were confirmed by prescriptions for ≥ 2 anti-TB drugs during a 30-day period [10]. Anti-TB drugs included isoniazid, rifampicin, ethambutol, pyrazinamide, amikacin, kanamycin, streptomycin, quinolones, thioamide, cycloserine, and para-aminosalicylic acid. To identify trends in the annual incidence of active TB between 2008 and 2017, we identified active TB cases for each calendar year in the entire Korean population. After excluding participants who were prescribed anti-TB drugs with a diagnosis of TB disease between 2006 and 2007, only participants with TB identified in 2008 were considered newly diagnosed cases in 2008. For the subsequent calendar years (2009-2017), only participants with TB who had not received anti-TB treatment in the previous 2 years were considered newly diagnosed cases.

There is no agreement on definitions and little internationally comparable information on the incidence of disabilities [2] and no information on disability incidence in the NDRD as well. Therefore, we operationally defined TB incidence in the disability group for each calendar year as any instance where the first disability registration date was earlier than the new diagnosis date of TB cases among people with disabilities in the NDRD. We also defined TB incidence in the disability group as occurring when the interval between these 2 dates was within 12 months, because it normally takes more than a year to register after disability incidence.

### Independent variables

We collected data on factors that may influence the incidence of active TB, such as sex, age, income level, and place of residence. As a proxy measure for actual household income, we used insurance premium quartiles-first quartile (lowest), second quartile, third quartile, and fourth quartile (highest)-and the category of Medical Aid beneficiaries, as provided by the Korean National Health Insurance Service. The first quartile of income level included Medical Aid beneficiaries. Insurance premiums are calculated based on the income, property, and automobile taxes for each household. Residential areas were grouped into 3 categories (metropolitan, city, and rural) based on the Korean ZIP code.

### Statistical analysis

In this serial cross-sectional study, we calculated the crude incidence rate of active TB for each calendar year from 2008 to 2017, plotted the annual incidence rates, and assessed the annual incidence trends between people with and without disabilities. The denominator of the annual incidence rate was defined as all the participants of the NHID identified in the corresponding year. The numerator was defined as those with active TB incidence in the same calendar year. The incidence rate of active TB was expressed as the number of active TB cases per 100,000 person-years. The age-standardized and sex-standardized incidence rate of active TB was also calculated using the direct standardization method. As subgroup analyses, we calculated the age-standardized and sex-standardized incidence rates of active TB according to the severity and type of disability. The NDRD defines 15 categories of disabilities. Disability severity is officially graded from 1 (very severe) to 6 (very mild) based on functional losses and clinical impairment, as determined by a medical specialist. In this study, disability severity was classified as severe (grades 1-3) or mild (grades 4-6).

Using data from all participants registered with the NHID in 2017, the most recent data available in our study, we described the general characteristics of the participants as follows. First, we compared the independent variables of people with and without disabilities in the 2017 data using descriptive statistics. Second, we identified the incidence of active TB and calculated the incidence rates among individuals with and without disabilities. Third, we stratified people with disabilities by severity and type and calculated the incidence rates in those groups. Fourth, we conducted a cross-sectional study to evaluate associations between disabilities and the development of active TB disease and performed logistic regression analysis using the 2017 data. We also developed a multivariable logistic regression model adjusted for age, sex, income level, and residence. In addition, multivariable logistic regression analysis stratified by severity and type of disability was performed. We conducted another subgroup analysis to assess the association between disability and TB incidence according to age (16-64 and ≥ 65 years). Unknown data were regarded as missing values. All analyses were performed using the SAS version 9.4 (SAS Institute Inc., Cary, NC, USA). Two-sided p-values of 0.05 were considered to indicate statistical significance.

### Ethics statement

This study was conducted in accordance with the Ethical Principles for Medical Research Involving Human Subjects, as outlined in the Declaration of Helsinki. The study protocol was approved by the International Review Board (IRB) of Chungbuk National University (IRB No. CBNU-202010-HRHR-0171). The requirement for informed consent was waived by the IRB because no patients were at risk.

## RESULTS

**Figure 1.**
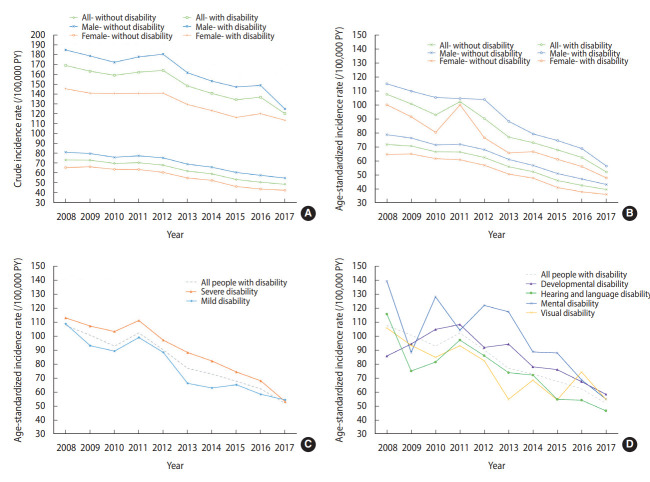
Trends in tuberculosis incidence between 2008 and 2017 among participants with or without disabilities. (A) Crude rates stratified by sex. (B) Age- and sex-standardized rates stratified by sex. (C) Age- and sex-standardized rates stratified by severity of disability. (D) Age- and sex-standardized rates stratified by type of disability. PY, person-years.

The total number of enrolled participants with disabilities increased from 2,338,534 in 2008 to 2,627,365 in 2017 (Supplementary Material 1). The overall incidence of active TB among the populations with and without disabilities decreased between 2008 and 2017 (Figure 1A). The crude incidence rates of active TB among people with disabilities were significantly higher than those among people without disabilities throughout all the observed years, regardless of sex (p<0.001) (Supplementary Material 2). The age-standardized and sex-standardized incidence rates of active TB among people with and without disabilities revealed patterns similar to their crude incidence rates (Figure 1B). The age- and sex-standardized rates decreased for both mild and severe disabilities, along with a decrease in the gap between those groups (Figure 1C). Despite the dispersed TB incidence rates among different types of disabilities in 2008, the age- and sex-standardized rates tended to decrease in 2017 (Figure 1D).

The most recent dataset, from the 2017 calendar year, was used to describe the baseline characteristics of the enrolled participants with and without disabilities (Table 1). In 2017, males were significantly more represented among people with disabilities (58.0 vs. 49.7%, p<0.001). Participants with disabilities were also older (60.5±18.0 vs. 40.0±21.0 years, p<0.001). The disability group was more likely to report lower income and live in rural areas. Among participants with disabilities, 38.4% had a severe form of disability, with the most frequent type being physical disabilities (50.2%).

In 2017, active TB incidence was detected in 3,150 (0.12%) participants with disabilities and 24,290 (0.05%) participants without disabilities (Table 2). People with disabilities had a higher crude incidence rate of active TB disease than those without disabilities (119.9/100,000 vs. 48.5/100,000 person-years, p<0.001), regardless of sex, income level, and place of residence. The crude incidence rate of active TB in elderly participants aged ≥ 80 years was higher than that in other age groups, and there was no difference in the crude incidence rate between people with and without disabilities (p=0.740) in the ≥ 80-year group. The crude incidence rates of active TB in people with mild and severe disabilities were 107.3 and 127.7 per 100,000 person-years, respectively (Table 3). Among the various types of disabilities, those due to respiratory disease had the highest crude incidence rate of active TB (234.5/100,000 person-years).

In a univariable logistic regression analysis, people with disabilities had higher odds of active TB incidence than those without disabilities (odds ratio [OR], 2.47; 95% confidence interval [CI], 2.38 to 2.57) (Table 4). Adjusting for sex, age, income level, and place of residence decreased the OR, but it still remained significant (adjusted OR [aOR], 1.19; 95% CI, 1.15 to 1.24). The association between severe disability and TB incidence was slightly stronger (aOR, 1.21; 95% CI, 1.14 to 1.29).

In the subgroup analysis, different types of disabilities revealed various associations with the incidence of TB. Participants with disabilities due to organ failure, such as those of the kidney, lung, heart, and liver, had high odds of developing active TB. The association between mental disabilities and TB incidence was significant in both univariable (OR, 2.21; 95% CI, 1.81 to 2.69) and multivariable analyses (aOR, 1.51; 95% CI, 1.24 to 1.84). Developmental disabilities were significantly associated with the incidence of TB after adjustment (aOR, 1.30; 95% CI, 1.09 to 1.55). Another subgroup analysis stratified by age showed that the association between disability and TB incidence was stronger in the young age group (aOR, 1.35; 95% CI, 1.27 to 1.44) than in the older age group (aOR, 1.10; 95% CI, 1.05 to 1.16) (Table 5).

## DISCUSSION

To the best of our knowledge, this is the first study to report that people with disabilities are at a higher risk of contracting TB disease. Although disabilities have been assumed to be a risk factor for TB disease owing to its relationship with low socioeconomic status and limited access to health care, no studies have investigated the association between disabilities and the subsequent risk of TB. We also identified several types of disabilities that are closely associated with TB. For example, people with mental and developmental disabilities are more prone to TB disease, which highlights the need for special attention and regular TB screening in these subgroups. In addition, we need to address various barriers that people with disabilities encounter while seeking care and adhering to treatment.

Despite global efforts to fight TB, the distribution of TB cases is unequal worldwide, with cases continuing to cluster among disadvantaged groups, such as the poor and minorities. A growing consensus indicates that progress in TB control requires action to address the social determinants of TB, along with investments in strengthening TB control programs. Weak social and economic policies and rapid urbanization are major upstream social determinants of TB epidemiology. These conditions give rise to an unequal distribution of social determinants of health, including food insecurity and malnutrition, poor housing and environmental conditions, and financial and cultural barriers to healthcare access. Disabilities and TB infection share these common social determinants of health, which generates a vicious cycle of disabilities and TB infection. The provision of public health interventions targeting disabilities and TB infection is only possible when we understand their links.

People from groups of low socioeconomic status are more likely to have more frequent contact with people with infectious TB [11]. Poverty and disabilities are believed to occur in a cycle [4]. Disabilities may lead to a lower standard of living and poverty through a lack of access to education and employment and through increased expenditures related to disabilities. Food insecurity and malnutrition are indirect poverty markers. The association between TB and undernutrition is well known [12]. Undernutrition diminishes one’s immune system, which increases the likelihood of developing active TB disease when infected with *Mycobacterium tuberculosis* [13]. Individuals with disabilities often face significant issues related to malnutrition [14,15]. These shared features of poverty and undernutrition are causes of TB risk among people with disabilities.

Urbanization and population growth have led to increased population density and crowded living and working conditions. Households with a low socioeconomic status generally experience poorer indoor air quality [16]. These populations are likely at risk of developing TB due to weakened host defenses against the disease. Poor ventilation and overcrowding increase the likelihood of uninfected individuals being exposed to TB. Both indoor [17] and outdoor [18] air pollution are risk factors for developing TB. People with disabilities are more likely to face poor housing and work environments. For example, the levels of exposure to outdoor air pollution among children with intellectual disabilities are significantly higher than those among families of children without intellectual disabilities [19]. The association between disabilities and these social determinants and their effects on TB disease are another important agenda for future studies.

People with disabilities are at risk of developing chronic conditions, which are called secondary conditions [20] and also constitute risk factors for TB disease. They are more likely to experience unhealthy behaviors and risk factors for chronic diseases, such as an unhealthy diet, high blood pressure, obesity, and limited physical activity. These health risk behaviors increase the risk of diabetes among people with disabilities [21,22]. Diabetes, in turn, increases the risk of developing TB by approximately 3 times, doubles the risk of death during TB treatment, and increases the risk of other poor TB treatment outcomes [23]. Since multimorbidity is frequently observed among people with disabilities, active case finding for TB disease would be beneficial to those with chronic diseases, such as diabetes.

The annual TB incidence among people with and without disabilities decreased from 2008 to 2017. This could be ascribed to the continued efforts to fight TB in Korea during the last decade. We observed that the disabled also benefited from Korea’s successful national TB control programs, resulting in decreasing active TB incidence among people with disabilities. It is noteworthy that the TB incidence among people with disabilities suddenly rose between 2011 and 2012. This date coincides with the implementation of the national personal assistance service for people with disabilities, although the linkage between these 2 events cannot be directly confirmed due to a lack of evidence. However, given that the gastric cancer screening rate rose sharply in 2022 among people with severe disabilities who were the target population of the national personal assistant service, it would be reasonable to assume that the service had the effect [24]. In addition, the high proportion of the elderly among females with disabilities could account for differences in the age-standardized rates between sexes and its sharp increase among females with disabilities.

People with disabilities often face barriers in reaching TB care services [25]. A lack of information and knowledge about TB disease affects health-seeking behaviors and delays initial contact with the healthcare system. The pathways from screening to completing anti-TB treatment are more challenging. These issues apply not only to the disabled, but also to various vulnerable groups that are at risk of developing TB disease. Delays in TB diagnosis and treatment consequently impose a high risk of unfavorable outcomes [26]. The WHO’s End TB Strategy highlights the importance of patient-centered care, which involves systematically assessing and addressing patients’ needs and expectations [1]. Educational, emotional, and social support are essential for patients to complete diagnostic process and full course of anti-TB treatment. This approach is possible only with the collaboration of relevant governments, healthcare providers, components of civil society, and communities, such as public-private partnerships [27]. When preparing national TB control strategies, this approach to integrated, patent-centered care and prevention, especially for vulnerable populations, such as the disabled, should be considered.

Age is an important determinant of developing TB disease and shaping TB epidemiology [28]. Our subgroup analysis revealed that an association between disability and TB incidence was stronger among those aged between 16 years and 64 years than among the elderly population (aged 65 years and older). Our result implies that elderly people without disabilities possess similar risks of developing TB disease, to those with disabilities. This result can be explained by the fact that elderly individuals are more likely to have chronic comorbidities, which weaken the immune system and increase the likelihood that a latent TB infection progresses to active TB disease. It is important to include the elderly with fragility and comorbidities in definitions of vulnerable populations.

Our study has some limitations. First, because of the operational definition of active TB cases using ICD codes and prescriptions of anti-TB drugs, we could not capture patients who died without visiting hospitals or initiating anti-TB treatment in this study. In Korea, people with disabilities still have poor access to healthcare services [29], which might delay TB diagnosis and treatment and impose greater risks of mortality. People with severe disabilities who developed TB but could not visit hospitals for further management might not have been included in the study population. This would have caused an underestimation of the incidence of TB among people with disabilities. Second, our results cannot be applied to other settings with different healthcare systems. The welfare strategy and characteristics of disabled individuals in other countries are different from those in Korea; thus, more evidence from multi-ethnic studies is required to determine TB incidence among people with disabilities. Third, information on clinical and demographic variables that may influence TB progression was not available from the NHID.

The results of this study have several important implications. One of the strengths of this study is its large scale based on a national healthcare claims database in Korea. Second, no previous study has attempted to evaluate long-term trends in TB incidence among people with disabilities or provided a detailed analysis according to the grade and type of disability. Third, as opposed to other studies that used self-report questionnaires about disability, we used data from a national registry, which is an objective and reliable way to collect information.

In conclusion, our study revealed that people with disabilities are at greater risk of developing TB disease. Mental and developmental disabilities were significantly associated with TB. It is essential to actively screen for TB cases among people with disabilities who are at risk of developing TB to achieve the goals of the WHO’s End TB Strategy. Understanding the association between disabilities and TB is important for identifying barriers to TB services and preparing TB health policies and programs that target people with disabilities [30].

## Figures and Tables

**Figure f2-epih-44-e2022098:**
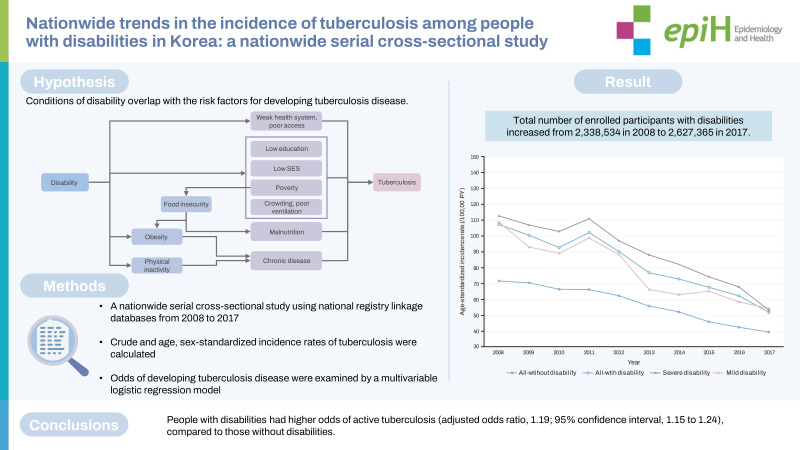


**Table 1.** Characteristics of enrolled participants with and without disabilities registered in 2017

Characteristics		All participants (n=52,712,239)	With disabilities (n=2,627,365)	Without disabilities (n=50,084,874)	p-value
Sex					
	Male	26,390,827 (50.1)	1,524,558 (58.0)	24,866,269 (49.7)	<0.001
	Female	26,321,412 (49.9)	1,102,807 (42.0)	25,218,605 (50.4)	
Age, Mean±SD (yr)		41.0±21.3	60.5±18.0	40.0±21.0	<0.001
	<30	16,840,651 (31.9)	184,853 (7.0)	16,655,798 (33.3)	<0.001
	30-39	7,634,258 (14.5)	143,358 (5.5)	7,490,900 (15.0)	
	40-49	8,788,690 (16.7)	286,639 (10.9)	8,502,051 (17.0)	
	50-59	8,583,409 (16.3)	510,958 (19.5)	8,072,451 (16.1)	
	60-69	5,723,391 (10.9)	572,848 (21.8)	5,150,543 (10.3)	
	70-79	3,423,916 (6.5)	578,206 (22.0)	2,845,710 (5.7)	
	≥80	1,717,924 (3.3)	350,503 (13.3)	1,367,421 (2.7)	
Income level (quartile)					
	First	10,737,721 (20.4)	939,380 (35.8)	9,798,341 (19.6)	<0.001
	Second	10,521,266 (20.0)	397,980 (15.2)	10,123,286 (20.2)	
	Third	13,281,795 (25.2)	519,345 (19.8)	12,762,450 (25.5)	
	Fourth	17,363,650 (32.9)	747,816 (28.5)	16,615,834 (33.2)	
	Unknown	807,807 (1.5)	22,844 (0.90)	784,963 (1.6)	
Residence					
	Metropolitan	32,887,108 (62.4)	1,437,919 (54.7)	31,449,189 (62.8)	<0.001
	City	15,279,505 (29.0)	818,874 (31.2)	14,460,631 (28.9)	
	Rural	4,519,094 (8.6)	370,566 (14.1)	4,148,528 (8.3)	
	Unknown	26,532 (0.1)	6 (0.0)	26,526 (0.1)	

Values are presented as number (%). SD, standard deviation.

**Table 2.** Characteristics and incidence of active tuberculosis among participants with and without disabilities in 2017

Characteristics		With disabilities (n=2,627,365)			Without disabilities (n=50,084,874)			p-value
		n (%)	PY	IR^1^	n (%)	PY	IR^1^	
All		3,150 (0.12)	2,627,365	119.9	24,290 (0.05)	50,084,874	48.5	<0.001
Sex								
	Male	1,901 (0.12)	1,524,558	124.7	13,608 (0.05)	24,866,269	54.7	<0.001
	Female	1,249 (0.11)	1,102,807	113.3	10,682 (0.04)	25,218,605	42.4	<0.001
Age (yr)								
	<30	44 (0.02)	184,853	23.8	2,840 (0.02)	16,655,798	17.1	0.030
	30-39	72 (0.05)	143,358	50.2	2,598 (0.03)	7,490,900	34.7	0.002
	40-49	182 (0.06)	286,639	63.5	3,484 (0.04)	8,502,051	41.0	<0.001
	50-59	468 (0.09)	510,958	91.6	4,578 (0.06)	8,072,451	56.7	<0.001
	60-69	647 (0.11)	572,848	112.9	3,825 (0.07)	5,150,543	74.3	<0.001
	70-79	933 (0.16)	578,206	161.4	3,869 (0.14)	2,845,710	136.0	<0.001
	≥80	804 (0.23)	350,503	229.4	3,096 (0.23)	1,367,421	226.4	0.740
Income level (quartile)								
	First	1,147 (0.12)	939,380	122.1	6,032 (0.06)	9,798,341	61.6	<0.001
	Second	464 (0.12)	397,980	116.6	5,158 (0.05)	10,123,286	51.0	<0.001
	Third	596 (0.11)	519,345	114.8	5,702 (0.04)	12,762,450	44.7	<0.001
	Fourth	914 (0.12)	747,816	122.2	7,075 (0.04)	16,615,834	42.6	<0.001
	Unknown	29 (0.13)	22,844	126.9	323 (0.04)	784,963	41.1	<0.001
Residence								
	Metropolitan	1,548 (0.11)	1,437,919	107.7	14,241 (0.05)	31,449,189	45.3	<0.001
	City	1,044 (0.13)	818,874	127.5	7,067 (0.05)	14,460,631	48.9	<0.001
	Rural	558 (0.15)	370,566	150.6	2,973 (0.07)	4,148,528	71.7	<0.001

PY, person-years; IR, incidence rate.

1 IR per 100,000 PY.

**Table 3.** Incidence of active TB stratified by grade and type of disability among participants with disabilities registered in 2017

Variables		With disabilities n (col %)	Active TB n (row %)	PY	IR
All		2,627,365 (100)	3,150 (0.12)	2,627,365	119.9
Severity of disability					
	Mild	1,618,856 (61.60)	1,082 (0.11)	1,008,509	107.3
	Severe	1,008,509 (38.40)	2,068 (0.13)	1,618,856	127.7
Type of disability					
	Physical disability	1,319,712 (50.20)	1,532 (0.12)	1,319,712	116.1
	Brain injury	258,610 (9.80)	257 (0.10)	258,610	99.4
	Facial disability	2,725 (0.10)	1 (0.04)	2,725	36.7
	Visual disability	259,423 (9.90)	319 (0.12)	259,423	123.0
	Hearing and language disability	327,694 (12.50)	558 (0.17)	327,694	170.3
	Developmental disability^1^	226,281 (8.60)	122 (0.05)	226,281	53.9
	Mental disability	91,560 (3.50)	98 (0.11)	91,560	107.0
	Renal disease	85,416 (3.30)	168 (0.20)	85,416	196.7
	Heart disease	7,181 (0.30)	13 (0.18)	7,181	181.0
	Respiratory disease	12,795 (0.50)	30 (0.23)	12,795	234.5
	Liver disease	12,246 (0.50)	17 (0.14)	12,246	138.8
	Ostomy	16,456 (0.60)	28 (0.17)	16,456	170.2
	Epilepsy	7,266 (0.30)	7 (0.10)	7,266	96.3

TB, tuberculosis; PY, person-years; IR, incidence rate.

1 Developmental disabilities included intellectual disability and autism.

**Table 4.** Logistic regression analysis of disability as a predictor of active tuberculosis incidence in 2017^1^

Variables		Model 1	Model 2	Model 3
		OR (95% CI)	aOR (95% CI)	aOR (95% CI)
Status of disability				
	Without disability	1.00 (reference)	1.00 (reference)	1.00 (reference)
	With disability	2.47 (2.38, 2.57)	1.23 (1.18, 1.28)	1.19 (1.15, 1.24)
Severity of disability				
	Without disability	1.00 (reference)	1.00 (reference)	1.00 (reference)
	Mild disability	2.64 (2.52, 2.76)	1.21 (1.15, 1.26)	1.19 (1.13, 1.24)
	Severe disability	2.21 (2.08, 2.35)	1.28 (1.20, 1.36)	1.21 (1.14, 1.29)
Type of disability				
	Without disability	1.00 (reference)	1.00 (reference)	1.00 (reference)
	Physical disability	2.40 (2.28, 2.52)	1.17 (1.11, 1.23)	1.14 (1.08, 1.20)
	Brain injury	2.05 (1.81, 2.32)	0.92 (0.81, 1.04)	0.90 (0.80, 1.02)
	Facial disability	0.76 (0.11, 5.37)	0.54 (0.08, 3.80)	0.51 (0.07, 3.60)
	Visual disability	2.54 (2.27, 2.83)	1.19 (1.06, 1.33)	1.15 (1.03, 1.29)
	Hearing and language disability	3.52 (3.23, 3.82)	1.30 (1.19, 1.42)	1.28 (1.18, 1.39)
	Developmental disability^2^	1.11 (0.93, 1.33)	1.47 (1.23, 1.76)	1.30 (1.09, 1.55)
	Mental disability	2.21 (1.81, 2.69)	1.72 (1.41, 2.10)	1.51 (1.24, 1.84)
	Renal disease	4.06 (3.49, 4.73)	2.20 (1.89, 2.56)	2.16 (1.85, 2.51)
	Heart disease	3.74 (2.17, 6.44)	1.79 (1.04, 3.08)	1.77 (1.03, 3.06)
	Respiratory disease	4.84 (3.38, 6.93)	1.96 (1.37, 2.81)	1.91 (1.33, 2.73)
	Liver disease	2.87 (1.78, 4.61)	1.74 (1.08, 2.80)	1.74 (1.08, 2.79)
	Ostomy	3.51 (2.42, 5.09)	1.34 (0.93, 1.94)	1.33 (0.92, 1.93)
	Epilepsy	1.99 (0.95, 4.17)	1.66 (0.79, 3.49)	1.50 (0.72, 3.15)

OR, odds ratio; aOR, adjusted odds ratio; CI, confidence interval.

1 The second multivariable logistic regression model was adjusted for age (continuous) and sex; The third multivariable model was adjusted for age (continuous), sex, income level, and place of residence.

2 Developmental disabilities included intellectual disability and autism.

**Table 5.** Logistic regression analysis of disabilities as a predictor of active tuberculosis incidence stratified by 2 age groups (16-64 and 65 years and older) in 2017^1^

Variables			Model 1	Model 2	Model 3
			OR (95% CI)	aOR (95% CI)	aOR (95% CI)
Age, 16-64 (yr)					
	Status of disability				
		Without disability	1.00 (reference)	1.00 (reference)	1.00 (reference)
		With disability	1.87 (1.76, 1.99)	1.47 (1.38, 1.56)	1.35 (1.27, 1.44)
	Severity of disability				
		Without disability	1.00 (reference)	1.00 (reference)	1.00 (reference)
		Mild disability	1.89 (1.74, 2.05)	1.39 (1.28, 1.51)	1.32 (1.22, 1.43)
		Severe disability	1.85 (1.69, 2.03)	1.57 (1.43, 1.72)	1.39 (1.26, 1.52)
Age, ≥65 (yr)					
	Status of disability				
		Without disability	1.00 (reference)	1.00 (reference)	1.00 (reference)
		With disability	1.24 (1.18, 1.30)	1.12 (1.06, 1.17)	1.10 (1.05, 1.16)
	Severity of disability				
		Without disability	1.00 (reference)	1.00 (reference)	1.00 (reference)
		Mild disability	1.25 (1.18, 1.32)	1.13 (1.07, 1.19)	1.11 (1.05, 1.18)
		Severe disability	1.23 (1.13, 1.33)	1.09 (1.00, 1.18)	1.07 (0.99, 1.16)

OR, odds ratio; aOR, adjusted odds ratio; CI, confidence interval.

1 The second multivariable logistic regression model was adjusted for age (continuous) and sex; The third multivariable model was adjusted for age (continuous), sex, income level, and place of residence.

## SUPPLEMENTARY MATERIALS

Supplementary Material 1. Flowchart of enrolled participants with and without disabilities between 2008 and 2017

Supplementary Material 2 Incidence of active tuberculosis among participants with and without disabilities stratified by sex between 2008 and 2017
