# Supplementary material for: Nationwide trends in the incidence of tuberculosis among people with disabilities in Korea: a nationwide serial cross-sectional study
Source: Epidemiol Health. 2022 Oct 28;44:e2022098. doi: 10.4178/epih.e2022098 (PMC10106551; doi:10.4178/epih.e2022098)
Supplement: Supplementary Material 1. — Flowchart of enrolled participants with and without disabilities between 2008 and 2017 [file epih-44-e2022098-Supplementary-1.docx]

**Supplementary Material 1.** Flowchart of enrolled participants with and without disabilities between 2008 and 2017

**
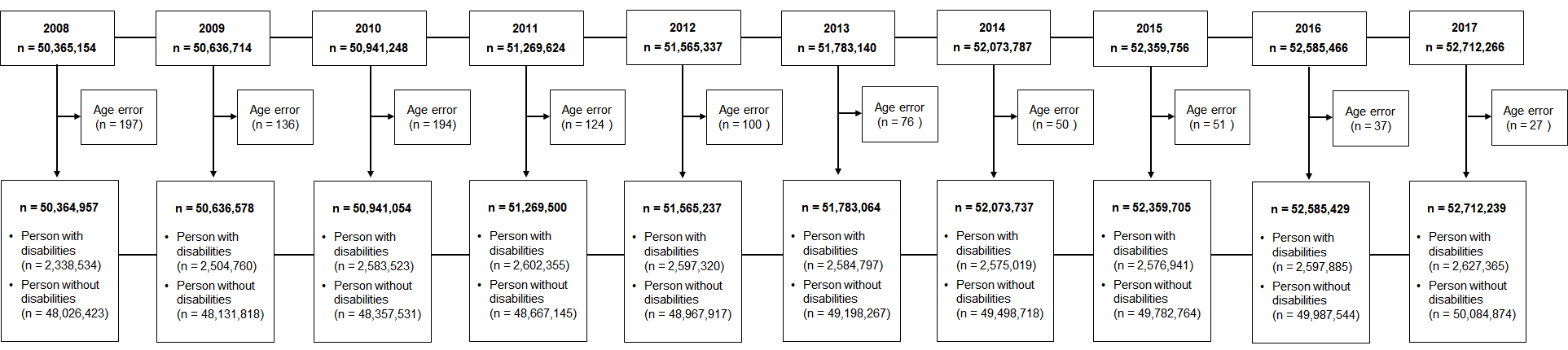
**
