# Supplementary material for: Nationwide trends in the incidence of tuberculosis among people with disabilities in Korea: a nationwide serial cross-sectional study
Source: Epidemiol Health. 2022 Oct 28;44:e2022098. doi: 10.4178/epih.e2022098 (PMC10106551; doi:10.4178/epih.e2022098)
Supplement: Supplementary Material 2 — Incidence of active tuberculosis among participants with and without disabilities stratified by sex between 2008 and 2017 [file epih-44-e2022098-Supplementary-2.docx]

**Supplementary Material 2.** Incidence of active tuberculosis among participants with and without disabilities stratified by sex between 2008 and 2017

| Calendar year | **All** | | | **Male** | | | **Female** | | |
| --- | --- | --- | --- | --- | --- | --- | --- | --- | --- |
|  | With  disability | Without  disability | P-value | With  disability | Without  disability | P-value | With  disability | Without  disability | P-value |
|  | IR | IR |  | IR | IR |  | IR | IR |  |
| 2008 | 168.7 | 73.1 | <0.001 | 184.4 | 80.8 | <0.001 | 145.1 | 65.4 | <0.001 |
| 2009 | 162.9 | 72.8 | <0.001 | 178.3 | 79.5 | <0.001 | 140.6 | 66.2 | <0.001 |
| 2010 | 158.8 | 69.6 | <0.001 | 171.9 | 75.7 | <0.001 | 140.3 | 63.6 | <0.001 |
| 2011 | 162.0 | 70.3 | <0.001 | 177.4 | 77.2 | <0.001 | 140.4 | 63.4 | <0.001 |
| 2012 | 163.6 | 67.8 | <0.001 | 180.0 | 75.2 | <0.001 | 140.7 | 60.4 | <0.001 |
| 2013 | 147.9 | 61.8 | <0.001 | 161.3 | 68.8 | <0.001 | 129.2 | 54.8 | <0.001 |
| 2014 | 140.3 | 59.0 | <0.001 | 152.8 | 65.7 | <0.001 | 123.0 | 52.4 | <0.001 |
| 2015 | 134.0 | 53.2 | <0.001 | 147.0 | 60.4 | <0.001 | 116.0 | 46.2 | <0.001 |
| 2016 | 136.6 | 50.5 | <0.001 | 148.5 | 57.5 | <0.001 | 119.9 | 43.6 | <0.001 |
| 2017 | 119.9 | 48.5 | <0.001 | 124.7 | 54.7 | <0.001 | 113.3 | 42.4 | <0.001 |

IR, incidence rate

Incidence rate per 100,000 person-years
